# A Systematic Review of the Audiological Efficacy of Cartilage Conduction Hearing Aids and the Factors Influencing Their Clinical Application

**DOI:** 10.3390/audiolres13040055

**Published:** 2023-08-10

**Authors:** Bei Li, Sinyoung Lee, Zuwei Cao, Takuji Koike, Robin Joseph, Tamsin Holland Brown, Fei Zhao

**Affiliations:** 1 Centre for Speech and Language Therapy and Hearing Science, Cardiff School of Sport and Health Sciences, Cardiff Metropolitan University, Cardiff CF5 2YB, UK; libei@nsmc.edu.cn; 2 Department of Otolaryngology, Affiliated Hospital of North Sichuan Medical College, Nanchong 637000, China; 3 Department of Mechanical Engineering, Faculty of Engineering, Graduate Faculty of Interdisciplinary Research, University of Yamanashi, Yamanashi 400-8511, Japan; leesinyoung@yamanashi.ac.jp; 4 Centre for Rehabilitative Auditory Research, Guizhou Provincial People’s Hospital, Guiyang 550002, China; bx430@126.com; 5 Department of Mechanical and Intelligent Systems Engineering, Graduate School of Informatics and Engineering, The University of Electro-Communications, Chofu 182-8585, Japan; t.koike@uec.ac.jp; 6 King Edward VII Hospital, Berkshire NHS Foundation Trust, Winsor SL4 3DP, UK; piousjosephrobin8@gmail.com; 7 Cambridgeshire Community Services NHS Trust, Cambridge CB1 3DF, UK; drtamsinhollandbrown@gmail.com

**Keywords:** cartilage conduction, bone conduction hearing devices, atresia, stenosis, conductive hearing loss, hearing aids

## Abstract

This systematic review evaluates the efficacy and benefit of cartilage conduction hearing aids (CC-HAs) and that factors that influence purchasing decisions. The hearing thresholds and functional gain following CC-HA wear were synthesised. A one-way analysis of variance compared the differences in the hearing thresholds and functional gain at individual frequencies and in patients with a variety of pathological changes. The synchronised aided hearing threshold and functional gain at 2.0 kHz were significantly better than at 0.5, 1.0, and 4.0 kHz. There was no significant difference in the synchronised unaided hearing thresholds across individual frequencies between 0.5 and 4.0 kHz. The synchronised functional gain in patients with atresia was significantly greater than in patients with aural atresia or stenosis and middle ear pathologies with normal ear canals. The acceptability of CC-HAs in terms of purchase decision is influenced by the condition of the external auditory meatus and severity of hearing loss, with the highest purchase rate seen in patients with aural atresia or stenosis. CC-HAs’ fitting procedure advantages and cosmetic considerations make these devices a viable and preferred choice for individuals with microtia and aural atresia. Additional research to evaluate the benefits towards emotional well-being is crucial to gain insights into the psychological impact of CC-HA use.

## 1. Introduction

Bone conduction (BC) is defined as the transmission of sound waves to the inner ear through the vibrating bones of the skull, bypassing the outer and middle ear, that enable the perception of sound. BC can be beneficial for patients with conductive hearing loss (CHL), as it allows for sound to be transmitted directly to the inner ear, instead of being conveyed through air conduction (AC) via the ear canal [1]. BC hearing aids are an established audiological intervention option for individuals with CHL. However, because of the disadvantages associated with traditional BC hearing aids, such as a limited frequency response range, sound distortion, discomfort, limited power, and cosmetic concerns, they have poor uptake and low acceptance in individuals with CHL [2,3,4].

With advances in technology, new and improved BC hearing devices (BCHDs) have been developed to address their disadvantages. For example, bone-anchored hearing aids (BAHAs) that use a new electronic signal transmission and an optimised transducer design to improve the bone conduction efficiency have been developed [3]. Evidence shows the audiological benefits of BAHAs in comparison to BC hearing aids and the resultant improvements in quality of life (QoL) [5]. BAHAs have become increasingly popular in recent years as effective rehabilitative tools for patients with CHL, mixed hearing loss, and single-sided deafness [3]. 

However, evidence shows that percutaneous BAHA (e.g., Baha^®^) has some reported drawbacks. There are high complication rates associated with abutment failure due to soft tissue infection from using a percutaneous and bone-anchored implant [3]. Transcutaneous BAHAs have been introduced as an alternative approach with the absence of a skin-penetrating abutment to mitigate such issues. These devices are placed under the intact skin, with an externally worn receiver that is connected magnetically to an internal component [6,7]. They are classified as passive and active depending on whether the vibrations are transmitted through the skin (passive transcutaneous skin-drive BCHDs, e.g., Baha^®^Attract) or directly into the bone (active transcutaneous direct-drive BCHDs, e.g., Bonebridge™). Apart from avoiding skin-penetration-related complications, the shift from percutaneous to transcutaneous sound transmission also enhances aesthetics. Comparing the hearing threshold improvement among devices, direct-drive BCHDs (i.e., percutaneous and active transcutaneous implanted transducers) provide greater gains compared to skin-drive BCHDs (i.e., BC hearing aids and passive transcutaneous implanted transducers) due to direct bone vibrations without the vibrations becoming attenuated through the soft skin [6,7].

Furthermore, a Vibrant Soundbridge (VSB) is another type of semi-implantable BC hearing device. It differs from the BAHA in terms of its surgical attachment to the ossicular chain and its direct stimulation of the inner ear through the mechanical vibration of the ossicles. Several recent studies have shown that the VSB has some advantages over the BAHA, such as better sound quality, feedback control, and appearance [8]. A recent review by Maw [8] suggests that the VSB provides a superior natural sound quality when compared to the BAHA, resulting in improved speech understanding and better sound perception in patients who have not had success with conventional AC hearing aids. However, both BAHAs and VSBs are expensive devices, and implantation requires complex surgery, which has a high risk of potential complications and subsequent costs [9]. 

**Figure 1:**
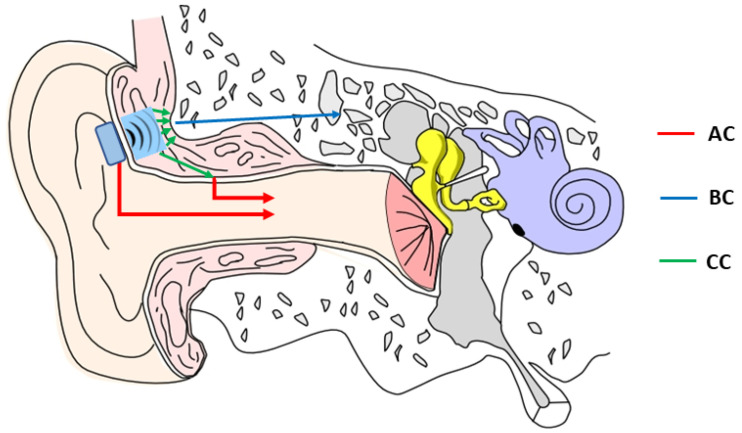
Diagram of the possible pathways associated with cartilage conduction.

A Japanese research group proposed a new concept with cartilage conduction as a third sound transmission pathway [10]. Its mechanism involves the transmission of sound through the cartilage and soft tissues of the outer ear, with the mechanical vibrations generated by the transducer propagated through the AC and BC pathways [11,12,13]. Figure 1 illustrates the possible pathways associated with cartilage conduction. 

Based on this sound transmission mechanism, a new cartilage conduction hearing aid (CC-HA) has been commercialised and used in clinical practice in Japan since 2017. It was shown to be a safe and effective alternative hearing rehabilitation tool for patients with hearing loss, particularly those who cannot use conventional AC hearing aids due to outer ear obstructions or malformations (e.g., atresia and microtia) [13]. However, a recent review by Nishimura et al. [12] highlighted the limitation in the current evidence of using CC-HAs in patients with hearing loss, such as the short duration of CC-HA use in clinical practice and the lack of comparative analyses with other hearing devices. Therefore, as a newly developed hearing device, there are many factors that might affect its performance and acceptability, such as the severity of hearing loss, functional gain, and self-perceived speech recognition improvement. These influencing factors need to be evaluated to facilitate further clinical application. This systematic review aims to assess the efficacy and benefits together with the factors influencing the purchase of a CC-HA. The outcome of this review will make a substantial contribution towards developing clear guidelines for the fitting of a CC-HA and will provide a valuable resource for healthcare professionals and patients in making informed decisions regarding the suitability of a CC-HA as a treatment option for hearing-impaired people in ENT/audiology clinics.

## 2. Literature Search Strategy

Taking into consideration the tracing of the evolutionary trajectory of any advancements and research associated with CC-HA, we decided to commence our search of the literature from the year 2004, spanning over the past two decades. As a result, PubMed and Cochrane were searched to identify relevant studies published from May 2004 to April 2023 using the following search terms: (1) “Hearing Loss, Conductive” OR “Conductive hearing Loss” OR “Conductive hearing impairment” OR “Conductive deafness” OR “Hearing Loss, Mixed” OR “Mixed hearing Loss” OR “Mixed hearing impairment” OR “Mixed deafness” OR “Otoscleros*” OR “Otospongios*” OR “Aural atresia” OR “Tympanic membrane perforation” OR “Otitis media” OR “Cholesteatoma”; (2) “Bone conduction device” OR “Cartilage conduction hearing aid” OR “Cartilage conduction device” OR “Cartilage conduction”. In addition, we searched the bibliographies of all identified relevant publications and the bibliographies from the citations of relevant articles. The publication language was restricted to English. 

### 2.1. Selection of the Literature: Inclusion and Exclusion Criteria

The titles and abstracts of all the studies identified using the search strategy were then screened by three independent authors (BL, ZC, and FZ). Subsequently, the same three authors confirmed the full text of the selected papers and evaluated their eligibility. The articles were selected on the basis of the inclusion and exclusion criteria shown in Table 1. 

### 2.2. Data Extraction and Data Processing

Three authors (BL, SL, and ZC) independently used an agreed data collection form to extract the data from the included studies. The extracted data were the study authors, publication year, study site, study design, sample classification and size, and main audiological outcomes (unaided and aided hearing thresholds and functional gain and speech recognition scores). The extracted data were double checked for accuracy, and any disagreements after discussion were arbitrated by one of the other authors (FZ and TK).

### 2.3. Data Synthesis and Statistical Analysis

The hearing thresholds obtained from the individual studies were synthesised to calculate the combined means and Standard Deviations (SDs) using the formula coded by R in the website, which can be accessed via the following link: https://www.statstodo.com/CombineMeansSDs.php (accessed on 24 May 2023). A one-way analysis of variance (ANOVA) was performed to compare the differences in the hearing thresholds at individual frequencies and in patients with various pathological changes. The level of significance was set at the conventional 5% level in each case. 

## 3. Results

### 3.1. A summary of the Characteristics of the Included Studies

**Figure 2:**
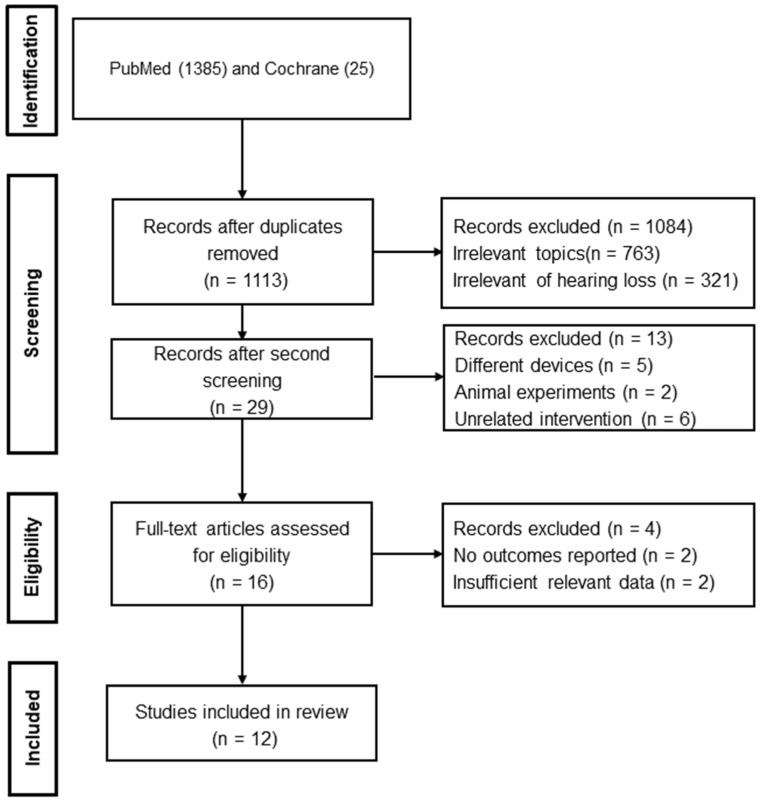
PRISMA flowchart of study selection.

According to the search strategy, 29 studies were retrieved, with 16 remaining after the removal of duplicates, irrelevant studies, and studies that did not meet the inclusion criteria. Of these, four studies were excluded after further examination because of insufficient outcomes reported. As a result, 12 eligible papers were included in the review (Figure 2).

A summary of the characteristics in the 12 included studies [14,15,16,17,18,19,20,21,22,23,24,25] is shown in Table 2. There was a total of 447 patients with various peripheral auditory disorders (excluding those from study [24] due to overlapping participant groups with study [20]). The age range of the patients ranged from newborns to individuals aged 90 years. The total number of male participants was 257 (57.5%), and the total number of female participants was 190 (42.5%). As shown in Table 2, the majority of studies (10/12, 83.3%) were conducted by several Japanese research groups, with one in Indonesia [21] and one in the USA [25]. As newly developed medical devices, the primary objective of the included studies was to explore the efficacy and benefits of CC-HAs in terms of functional gain and speech recognition scores together with user acceptability. In addition, Suwento et al. [21] conducted a study to assess the impact and satisfaction of CC-HAs in children and young adults with microtia and aural atresia, while Nairn et al. [25] compared the audiometric outcomes of CC-HAs with traditional BCHDs. 

The first clinical trial result was from a case study conducted by Hosoi et al. [14]. It indicates that the optimal position for the cartilage conduction transducer is the tragal cartilage. Moreover, the device provided the patient with better hearing ability than a traditional BC hearing aid. Building on this work, several Japanese research groups conducted a series of studies with the device to investigate the benefits for hearing-impaired patients with various pathological changes in the external auditory canal and/or middle ear structures. In the 12 included studies, five main classifications of ear pathology were clearly reported in 10 studies (all except Nishimura et al. [20] and Nishimura et al. [24]). These were atresia (211 ears), congenital stenosis (17 ears), stenosis post-surgery (19 ears), several types of middle ear disorders (e.g., otorrhea and chronic otitis media) (14 ears), cases associated with middle ear malformation (6 ears), and single-sided hearing loss (3 ears).

### 3.2. Summary of Audiological Characteristics and Efficacy of CC-HAs

In general, these studies show that the CC-HAs are efficacious in improving hearing thresholds, speech recognition, and overall auditory ability in adults and children with various pathologies of the ear, particularly aural atresia or stenosis.

#### 3.2.1. Severity of Hearing Loss in Patients Who Were Fitted with CC-HAs

**Figure 3:**
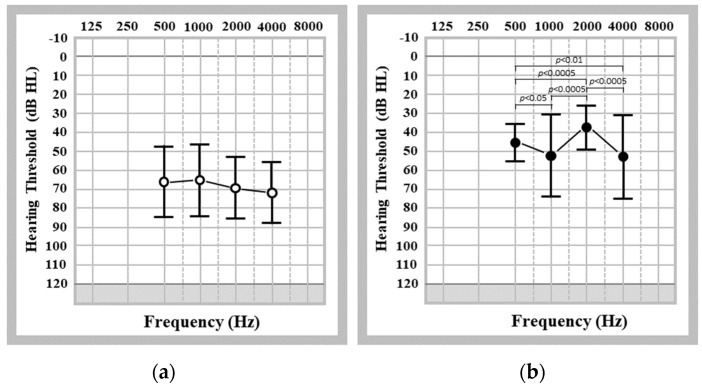
Synchronised unaided hearing thresholds across frequencies of 0.5–4.0 kHz. (**a**) Synchronised unaided hearing thresholds across individual frequencies between 0.5 and 4.0 kHz based on the data obtained from three studies [15,17,19]; (**b**) synchronised aided hearing thresholds across individual frequencies between 0.5 and 4.0 kHz based on the data obtained from three studies [15,17,19].

In the 12 studies included in this review, the averaged hearing thresholds across frequencies 0.5 to 4.0 kHz were presented in ten studies. The AC hearing thresholds ranged from 58.04 to 85.99 dB HL, whilst the BC hearing thresholds ranged from 8.3 to 49.12 dB HL. The average synchronised AC and BC thresholds were 69.30 dB HL (SD: 15.68 dB HL) and 18.19 (SD: 15.19 dB HL), respectively. Figure 3a shows the synchronised unaided hearing thresholds at frequencies from 0.5 to 4.0 kHz based on the data from three studies [15,17,19]. The one-way ANOVA showed no significant difference in the synchronised unaided hearing thresholds across individual frequencies between 0.5 and 4.0 kHz (F = 1.01, *p* > 0.05). 

#### 3.2.2. Aided Hearing Thresholds in Patients Who Were Fitted with CC-HAs

The aided hearing thresholds measured in the free field ranged from 27.5 to 55.95 dB HL, and the synchronised hearing threshold was 38.16 dB HL (SD: 13.50 dB HL). Figure 3b shows the aided hearing thresholds at frequencies from 0.5 to 4.0 kHz based on the same three studies. There was a significant difference in the synchronised aided hearing thresholds at individual frequencies between 0.5 and 4.0 kHz (one-way ANOVA: F = 11.84, *p* < 0.0005). A further analysis revealed that the aided hearing thresholds were significantly better at 2.0 kHz than at 0.5, 1.0 kHz, and 4.0 kHz (F_0.5 vs. 2 kHz_ = 15.30, F_1 vs. 2 kHz_ = 22.78, F_4 vs. 2 kHz_ = 27.47, *p* < 0.0005). The aided hearing thresholds at 0.5 kHz were also significantly better than the aided hearing thresholds at 1.0 and 4.0 kHz (F_0.5 vs. 1 kHz_ = 5.76, *p* < 0.05; F_0.5 vs. 4 kHz_ = 7.73, *p* < 0.01). However, there was no significant difference in the synchronised aided hearing thresholds between 1.0 and 4.0 kHz (F_1 vs. 4 kHz_ = 0.04, *p* > 0.05).

#### 3.2.3. Functional Gain Provided by CC-HAs

**Figure 4:**
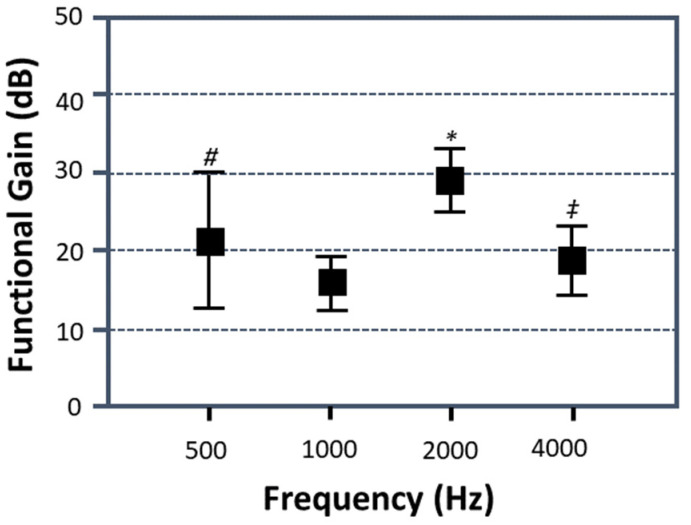
Synchronised functional gain across frequencies of 0.5–4.0 kHz. *: Synchronised functional gain at 2 kHz is significantly greater than the gains at 0.5, 1.0, and 4.0 kHz. #: Synchronised functional gain at 0.5 kHz is significantly greater than the gains at 1.0 and 4.0 kHz. *‡*: Synchronised functional gain at 4.0 kHz is significantly greater than that at 1.0 kHz.

Seven studies provided data on the functional gain provided by CC-HAs. The data show a functional gain between 16.66 and 49.00 dB, with an average synchronised gain at 29.27 dB (SD: 9.91 dB). Figure 4 shows the synchronised functional gain across individual frequencies based on the data obtained from same three studies. The highest gain was at 2.0 kHz (29.86 ± 4.12 dB), and the lowest gain was at 1.0 kHz (16.04 ± 3.52 dB). The statistical analysis showed a significant difference in the synchronised aided hearing thresholds at frequencies from 0.5 to 4.0 kHz (one-way ANOVA: F = 12.96, *p* < 0.0005). A further analysis revealed that the synchronised functional gain at 2.0 kHz was significantly higher than the gains at 0.5, 1.0, and 4.0 kHz (F_0.5 vs. 2 kHz_ = 68.53, F_1 vs. 2 kHz_ = 682.93, F_4 vs. 2 kHz_ = 318.90, *p* < 0.0005). The gains at 0.5 Hz and 4.0 kHz were also significantly greater than that at 1.0 kHz (F_0.5 vs. 1 kHz_ = 38.98, *p* < 0.0005; F_1 vs. 4 kHz_ = 27.43, *p* < 0.0005). Figure 4 also shows that the gain differed between 0.5 and 4.0 kHz (F_0.5 vs. 4 kHz_ = 8.55, *p* < 0.005).

Although there were no significant differences in the unaided hearing thresholds in the different categories of pathology, there were significant differences in the synchronised functional gains in aided patients with various ear canal pathologies (one-way ANOVA: F = 7.03, *p* < 0.005). The synchronised functional gain in patients with atresia was significantly greater than those with stenosis and middle ear pathologies but with normal ear canals (F_A vs. S_ = 6.29, *p* < 0.05; F_A vs. N_ = 10.80, *p* < 0.005) (Table 3). However, there was no significant difference in the gain between the stenosis group and the patients with normal ear canals (F_S vs. N_ = 0.54, *p* > 0.05). With the atresia or stenosis patients showing the higher gains, the results indicate that cartilage conduction is effective in improving the hearing ability in these two groups. 

### 3.3. Improvements in Speech Recognition Using CC-HAs

Akasaka et al. [18] examined the benefits of CC-HAs for speech perception in patients with unilateral aural atresia. Apart from an improvement in the aided hearing thresholds, their results show that the speech recognition scores at low speech levels were significantly improved. However, there was no significant improvement in the speech recognition score (SRS) in the presence of background noise. Suwento et al. [21] also showed that the speech recognition threshold and speech discrimination levels were significantly improved using CC-HAs in children and adolescents with atresia or microtia. The authors noted, however, that the benefits for speech perception were variable in these patients due to the nature of the damage to the auditory pathways (e.g., hair cells or auditory nerve damage).

### 3.4. Influencing Factors on the Benefits and Acceptability of CC-HAs

The various influencing factors on CC-HA performance and benefits have been explored, such as fixation and coupling methods of the transducer and the condition of the external ear canal that can influence the transmission efficacy of CC-HAs. Several studies show an improvement from the use of various tapes as a fixation method as they provide additional compression over the CC-HA transducer and are simple and safe to use [16,17,19]. Furthermore, the effect of applying coupling gel to the transducer of the CC-HA on aided thresholds and SRSs was explored by Nishimura et al. [19] Their results show that gel application significantly improved the aided thresholds at lower frequencies of 0.25 and 0.5 kHz, and particularly in patients who subjectively reported improvements. Although the application of a gel is considered a low-risk option that can enhance the benefits of CC-HAs, the subjective perception of their effectiveness was variable among patients, indicating that further objective investigations are needed to evaluate the signal quality and gains using computer simulations.

As shown in Table 2, several studies examined the factors that affect the purchase rate of CC-HAs in adult and paediatric patients with various ear conditions [17,19,24]. Nishimura et al. [17,19] showed that the overall willingness to purchase CC-HAs after a trial period was 60.47% in adults and 72.92% in children with hearing loss, and that the purchase decision was dependent on the condition of the patients’ external auditory meatus and the severity of their hearing loss. Patients with canal asteria or stenosis had the highest purchasing rate (81.25%), while patients with and without an abnormal canal had purchase rates of 47.06% and 50%, respectively [17].

A later study by Nishimura et al. [24] investigated the factors influencing the purchase rate of CC-HAs in 249 patients with varying ear conditions. The patients were divided into six groups, e.g., bilateral or unilateral closed or open ear canals and bilateral chronic continuous otorrhea that is unsuitable for conventional AC hearing aids. Age played a significant role in the purchase decision in the unilateral closed ear group, with younger individuals being more likely to make a purchase. In the bilateral closed ear canal group, those choosing to purchase had better-aided thresholds at 0.25 and 0.5 kHz compared to the non-purchasers. However, there were no significant differences in the functional gains and speech recognition scores between the purchase and non-purchase cases in all six groups. The type of transducer had an impact on the continued use rate, with the simple transducer type showing a lower rate in the bilateral chronic continuous otorrhea group and the hearing-impaired patients with bilateral or unilateral normal ear canal groups.

It is noteworthy that there were challenges in obtaining the desired gains as a result of the severity of hearing loss [16]. Limitations in the output level in some patients could preclude any significant improvements from using CC-HAs. In comparison to other BCHDs, Nairn et al. [25] suggested that there were approximately 5 dB differences in the aided hearing thresholds and functional gains between BCHDs and CC-HAs, with a favouring of BCHDs mainly in high frequency performance. 

## 4. Discussion

### 4.1. Cartilage Conduction: Mechanisms and Research Needs

The concept of cartilage conduction as the third sound transmission pathway, along with traditionally accepted AC and BC pathways, is of great interest. A recent review by Nishimura et al. [12] summarised the possible mechanisms of the cartilage conduction in terms of sound transmission, i.e., cartilage conduction utilises the vibration of the cartilaginous portion of the ear canal to aid in sound propagation to the ear canal (AC pathway) and skull bones (BC pathway) with the cartilaginous portion of the ear canal acting like the movable plate of a vibration speaker, providing signal amplification, as shown in Figure 1.

BC headphones, a commercial product that utilises Bluetooth to connect to mobile phones, have been specifically designed for users such as runners or cyclists. These headphones enable them to enjoy music or answer phone calls while keeping their ear canals open, ensuring they remain aware of their surroundings and minimizing the risk of accidents. Depending on the design, these headphones seem to be using similar cartilage conduction mechanisms for sound transmission, although different transducers are used in these two devices. 

Recently, an innovation report was published on the use of BC headphones as a part of a hearing assistant kit for children with glue ear, with the preliminary results showing some significant benefits [26]. This work also showed that BC headphones were able to improve children’s hearing during a crucial stage of their development via remote management during the COVID-19 pandemic. More importantly, BC headphones empowered parents and caregivers to support their children’s hearing needs, enhanced online learning clarity, and overcame difficulties caused by face masks that obstructed lip reading. Parental feedback indicated that children using the BC headphones along with the microphone and related app or online software experienced better hearing at home and at school, and some even showed improvements in pronunciation, behaviour, and listening anxiety. Therefore, the approach of using CC-HAs and BC headphones seems to provide an effective tool for people with hearing loss. 

However, it should be noted that there are limited studies available, and the concept and application have not yet been recognised widely in the professional community and by service users in comparison to other BC hearing devices. A further exploration of the mechanism of cartilage conduction is needed. Conducting theoretical investigations using a computer model analysis (e.g., finite element (FE)) would be a valuable approach to simulate and quantify the contribution of cartilage conduction as a third pathway for sound transmission. Such studies would help to determine the extent to which cartilage conduction influences the overall sound transfer function. The significant results derived from these studies provide valuable insights into the role of cartilage conduction in auditory function, and thus facilitate the applications of CC-HAs.

Furthermore, evidence has shown that measuring the effective gain is a useful approach for evaluating the effectiveness of sound transmission to cochlea for BAHA users, independent of the impact by the AC-BC gap [27,28]. The direct comparison of the difference between aided hearing thresholds and BC thresholds holds the potential to improve our understanding of the sound transmission mechanisms of the cartilage conduction, particularly in elucidating the contribution of sound transmission through the BC pathway in CC-HA users. Therefore, it would be important to incorporate measurements of effective gain in future studies. 

### 4.2. Advantages and Limitations of CC-HAs in Comparison to Current Commercially Available BC Hearing Devices

The successful application of CC-HAs in patients with various external ear canal and middle ear disorders is evident. There are benefits in terms of improving hearing ability and providing optimal hearing amplification, fitting procedure advantages, and cosmetic considerations. Consequently CC-HAs provide an advantageous choice for some patients with microtia and aural atresia as they may have reservations or preferences against surgical interventions like BAHAs. They may also be beneficial for patients who do not experience an improvement or amplification after undergoing ear reconstruction surgery or for patients with chronic continuous otorrhea who have not had success with conventional AC hearing aids. 

In comparison to other BCHDs, although there is a small advantage of BCHDs over CC-HAs, mainly in high-frequency performance, CC-HAs and BCHDs perform similarly in speech testing in most listening conditions [25]. In addition, the study by Nairn et al. [25] showed no clear preference in the sound localization and quality of life between these hearing devices and showed a higher purchase rate for CC-HAs. 

It is noteworthy that patients with congenital aural atresia could experience decisional conflict, which refers to the uncertainty, doubt, or difficulty in making a decision when faced with two or more options, each with potential benefits and drawbacks [29,30]. In the study by Graham et al. [31], the authors found that each intervention option (i.e., surgical intervention, traditional BC hearing aids, or BAHAs) had its own advantages and disadvantages for children with unilateral aural atresia. As a result, parents encountered several factors that led to decisional conflict, which were as follows:

(1) Potential risks such as surgical complications, device-related issues, and the need for ongoing maintenance and care;
(2) Long-term implications in terms of the child’s comfort, speech and language development, social interactions, and educational outcomes;
(3) Financial implications of device cost and ongoing expenses;
(4) Psychological impact such as the emotional distress associated with worrying about the potential impact of their decision on their child’s well-being and quality of life.

In regard to CC-HA fitting, the results obtained by Nishimura et al. [24] suggest that acceptability in terms of purchasing decision is affected not only by audiological factors (e.g., severity of hearing loss, subjective perception in hearing ability) and the type of transducers, but also by non-audiological-related factors (e.g., cosmetic consideration, user-friendliness, and affordability). Nishimura et al. [24] also found no significant differences in the functional gains and speech recognition scores between the purchasers and non-purchasers in any of the groups. Therefore, the high purchase rate in the bilateral closed ear canal group (i.e., aural atresia or microtia) could not be totally explained by better audiometric performances. 

A study by Li et al. [32] found that children between the ages of 8 and 13 who had microtia and aural atresia experienced a higher prevalence of social issues. These issues manifest in the form of interpersonal sensitivity, depression, anxiety, and hostility. This result indicates that these patients faced challenges in their social interactions. Additionally, the children with microtia and aural atresia were reluctant to participate in hearing tests due to underlying psychosocial problems, despite the parents expressing their willingness to proceed with the CC-HA fitting. 

### 4.3. Proposed Future Studies

Further development of CC-HA fitting algorithms to incorporate real-ear measurements (REMs) would provide benefits in terms of verifying the AC sound amplification in users with stenotic ear canals and those with normal ear canals. This approach facilitates essential adjustments to achieve optimal amplification, and thus maximise speech intelligibility. Moreover, in light of recent advancements within the hearing aid industry, together with new commercially available CC-HA models equipped with feedback suppression functions (i.e., HB-J1CC and HB-A2CC; Rion Co. Ltd., Kokubunji, Japan) [33], the incorporation of supplementary features into CC-HAs holds the potential to bring substantial benefits to users. For example, advanced signal processing for speech enhancement would improve sound clarity, and thus lead to enhanced speech recognition. The integration of wireless connectivity can enable users to connect with various electronics and other everyday wireless accessories, facilitated by a user-friendly setup.

Furthermore, additional research is necessary to measure and evaluate the subjective benefits and satisfaction of CC-HA use with relevant outcome measures, such as the International Outcome Inventory for Hearing Aids (IOI-HA; Satisfaction with Amplification in Daily Life (SADL)). Besides audiometric improvements, these questionnaires would provide standardised and validated methods to assess patients’ subjective experiences with CC-HAs, their perceived benefit, and overall satisfaction. In the meantime, to evaluate the psychological benefits of using hearing aids, several questionnaires have been developed, including the Psychosocial Impact of Assistive Devices Scale (PIADS) and the Glasgow Hearing Aid Benefit Profile (GHABP). In future studies, these questionnaires will help in gaining insights into the psychological impact and emotional well-being of individuals using CC-HAs. As a result, professionals can better understand the psychological effects of CC-HA use and address concerns such as emotional distress. This will enable them to optimise interventions and collaborate with patients in making informed decisions for a personalised and effective approach to their hearing healthcare.

## 5. Conclusions

This systematic review shows that CC-HAs appear to be effective hearing devices to improve hearing thresholds, speech recognition, and overall hearing ability in patients with various types of hearing loss. Both the synchronised aided hearing thresholds and functional gain at 2.0 kHz were significantly better than those at the frequencies of 0.5, 1.0, and 4.0 kHz. Furthermore, the synchronised functional gain in patients with atresia was significantly greater than in patients with stenosis and those with middle ear pathologies but with open ear canals. This suggests that CC-HAs may provide greater benefits for individuals with atresia. The acceptability in terms of purchasing the device is affected by the condition of the external auditory meatus and the severity of hearing loss. Patients with aural atresia or stenosis demonstrated the highest purchasing rate. Although CC-HAs do not demonstrate advantages in terms of functional gain in comparison to existing BCHDs, due to its fitting procedure advantages and cosmetic considerations, CC-HAs are shown as a viable and advantageous choice for some patients with aural atresia or microtia. Further research is needed to assess the subjective benefits and satisfaction of CC-HAs using objective outcome measures. This will provide insight into patients’ experiences, perceived benefits, and overall satisfaction as well as the psychological impact and emotional well-being of CC-HA users. Furthermore, an FE analysis and the inclusion of effective gain measurements in future studies would provide valuable insights into the role of cartilage conduction for sound transmission in auditory function and thus further facilitate the application of CC-HAs.

## Figures and Tables

**Table 1:** Summary of the key inclusion and exclusion criteria.

	Detailed Items
Inclusion criteria	Published within the past 15 years, in English Clinical trials Cartilage conduction hearing aids in patients with hearing loss Measuring efficacy and/or acceptability, safety, benefits, limitations, or patient-reported outcomes
Exclusion criteria	Reviewed articles, conference abstracts, editorials, opinion pieces, and letters to the editor Not a clinical study involving humans Different hearing devices or treatment methods Only recruited participants with sensorineural hearing loss Lacking sufficient information in evaluation of efficacy and/or acceptability Neither safety nor performance nor quality of life data reported

**Table 2:** Summary of the characteristics of the included studies in terms of the aim, study methods, and main outcomes.

Author, Year, and Country	Aim	Study Design, Demographic Data, Pathological Characteristics, and Audiological Measurements	Main Outcomes/Conclusions
1. Hosoi et al., 2010; Japan [14]	To examine the efficacy of a new sound conduction pathway via the cartilage.	A single-subject case study N * = 1 (male, 11 years old) Bilateral atresia (n * = 2) PTA (LE: AC/BC = 58.3/6.7 dB HL) Aided hearing thresholds in sound field	The tragal cartilage was the best position for the cartilage conduction transducer. The hearing improvement obtained by using CC-HAs is equivalent to that obtained with BC hearing aids.
2. Nishimura et al., 2013; Japan [15]	To investigate the benefits of cartilage conduction in hearing-impaired patients who cannot use air-conduction hearing aids due to continuous otorrhea or aural atresia.	A prospective cohort study N = 4 (male = 2; female = 2; age range: 25–73 years) Otorrhea/otitis media (n = 3); atresia (n = 1); stenosis/stenosis after surgery (n = 3); malformation (n = 1) PTA thresholds/aided hearing thresholds/gains in sound field at frequencies of 0.5–4.0 kHz	Hearing thresholds and speech recognition scores improved in all subjects. Unexpectedly large gains were obtained below 2 kHz in the patient with acquired aural atresia. The limitation of the output level is insufficient to provide the target gains for the patients with severe hearing loss.
3. Nishimura et al., 2017; Japan [16]	To investigate the benefits of CC-HAs in patients with severe conduction hearing loss and to evaluate its acceptability in clinical practice.	A prospective cohort study N = 41 (male = 26; female = 15; age range: 3–80 years) Bilateral atresia (N = 21); unilateral atresia (N = 15); other (N = 5) Aided hearing thresholds in sound field The benefits of the CC-HAs using the “Guidelines for evaluation of hearing aid fitting” published by the Japan Audiological Society	Hearing thresholds were significantly improved by using CC-HAs. The functional gains of using CC-HAs were approximately equivalent to previous hearing aids they used. The style of the transducer fixation and the type of aural atresia had no significant influence on the functional gains. The CC-HA use in most participants was sufficient or acceptable.
4. Nishiyama et al., 2021a; Japan [17]	To assess the efficacy of CC-HAs in adult hearing-impaired patients with abnormal or normal ear canal and determine who are good candidates for CC-HAs.	A cross-sectional design N = 37 (male = 17; female = 20; age range: 21–83 years) Atresia (n = 14); stenosis/stenosis after surgery (n = 11); otorrhea/otitis media (n = 8); otosclerosis/SHNL/single HL (n = 10) PTA thresholds/aided hearing thresholds/gains in sound field at frequencies of 0.5–4.0 kHz	CC-HAs provided hearing improvements in all frequencies. A total of 60.47% of all participants purchased a CC-HA after the trial. Of those, there was an 81.25% purchase rate in patients with asteria or stenosis. Significantly higher purchase rate in patients with mild hearing loss (85.71%) than those with severe hearing loss (20%) in the group of patients with asteria or stenosis.
5. Akasaka et al., 2021; Japan [18]	To evaluate the benefits of CC-HAs in terms of speech perception in patients with unilateral aural atresia.	A cross-sectional study N = 11 (male = 8; female = 3; age range: 7–83 years) Unilateral atresia (n = 11)∙ Aided hearing thresholds in sound field Speech recognition measurement using monosyllables test materials authorised by the Japan Audiological Society (i.e., 57-S or 67-S word lists) Levels of speech recognition test: dB max (the speech level at which the maximum score was obtained) and dB (max-10) (i.e., low speech level)	The speech recognition scores at low speech levels were significantly improved using CC-HAs. The speech recognition scores decreased under the presence of background noise. There was no significant difference in speech recognition scores between aided and unaided binaural hearing conditions in the presence of background noise.
6. Nishiyama et al., 2021; Japan [19]	To evaluate the efficacy of CC-HAs as well as the impact on hearing and security by incorporating additional tape compression over the transducer in children with hearing loss.	A prospective cohort study N = 42 (male = 27; female = 15; age range: 0–17 years) Bi-atresia (N = 8); uni-atresia (N = 27); bi-stenosis (N = 3); uni-stenosis (N = 2); Malformation (N = 1); single-sided hearing loss (N = 1) Pure-tone thresholds were obtained with over-ear headphones to assess AC (0.25 kHz–8 kHz) and BC (0.5 kHz–4.0 kHz) thresholds Sound-field thresholds were measured to assess CC-HA aided and unaided thresholds	Significant improvement in hearing thresholds at all frequencies by using CC-HAs in children with various types of hearing loss. Functional gains were improved mainly at low frequencies by applying additional tape compression over the CC-HA transducer. Additional tape compression over the CC-HA transducer was an easy and safe method for improving the hearing and stability of the CC-HA. A total of 72.92% of participants purchased a CC-HA after the trial.
7. Nishimura et al., 2021c; Japan [20]	To evaluate the understanding and acceptability of CC-HAs in clinical practice to share information about their medical applications, fitting methods, benefits, and alternatives as hearing aids for patients with hearing loss.	A survey study in nine medical institutions N = 256 (male = 144; female = 112; age range: 17–60 years) N = 93 (36%) previous users of hearing aids Bi-atresia (N = 65); uni-atresia (N = 124); uni-stenosis + HL with normal ear canal (N = 11); uni-HL with normal ear canal (N = 13); bi-otorrhea/otitis media (N = 9); bi-HL with normal ear canal (N = 27); single-sided hearing loss (N = 7) Aided hearing thresholds in sound field	Overall, 190 out of 256 participants (74%) purchased a CC HA. Higher purchase rates were found in the bi-closed and uni-closed ear (ear atresia or severe stenosis) groups (78–86%). The bilateral otorrhea group also had a high purchase rate (78%), although the functional gains of CC-HAs were poor at 4.0 kHz than the bi-closed ear group. Purchase rates of 37–57% were found in patients who felt that they benefitted from using conventional AC hearing aids.
8. Suwento et al., 2021; Indonesia [21]	To assess the outcomes and evaluate the impact and satisfaction of CC-HAs in children and young adults with microtia and aural atresia.	A quasi-experimental study design N = 10 (male = 4; female = 6; age range: 9–19 years) Bilateral microtia/atresia (N = 8); unilateral microtia/atresia (N = 2) A total of 14 ears from 8 patients were measured for unaided/aided hearing thresholds in sound field A total of 14 ears from 7 patients were assessed for speech recognition threshold (SRT) and speech discrimination score (SDS) Hearing aid adaptability questionnaire	Significant better aided hearing thresholds were found by using CC-HAs in 14 ears from 8 patients. Significant improvement in SRT and SDS with CC-HAs in 12 ears from 7 patients. Almost all parents reported satisfaction with the performance of CC-HAs, such as good adaptability, effectiveness, operability, appearance, and comfortability, after 6-month CC-HA use.
9. Nishimura et al., 2021d; Japan [22]	To investigate the impact of gel application on the efficacy of CC-HAs.	A quasi-experimental design N = 23 (male = 16; female = 7; age range: 20.6 ± 17.3 years) Atresia/stenosis (N = 23) Unaided/aided hearing thresholds in sound field Aided hearing thresholds in sound field Speech recognition measurement using 20 monosyllable test materials (i.e., 67-S word lists)	The aided thresholds were significantly improved with the application of gel for frequencies above 1 kHz. The aided thresholds in the improved patients with gel were significantly better than those in the unchanged patients. There was a significant difference between the dry and gel conditions in improved patients. The application of gel led to a decrease in dB (max) in improved patients compared to unchanged patients.
10. Kitama et al., 2022; Japan [23]	To evaluate the performance and subjective reported hearing improvement using CC-HAs, BAHAs, and ADHEAR systems to provide information for patients to select the most suitable BCHD.	A case–control study N = 6 (male = 3; female = 3; age range: 30–73 years) Atresia (n = 2); stenosis (n = 1); OMC (n = r); malformation/superior canal dehiscence syndrome (n = 3) Unaided/aided hearing thresholds in sound field The sound localization test was performed Glasgow Benefit Inventory questionnaire was used to evaluate the QoL	The gain of BAHAs and CC-HAs is greater than that of ADHEAR systems. There was no significant tendency of any of the hearing devices to help the sound localization. Regarding the Glasgow Benefit Inventory, there is no significant difference among CC-HAs, BAHAs, and ADHEAR systems in the QoL evaluation.
11. Nishimura et al., 2022; Japan [24]	To investigate factors influencing purchase rate of CC-HAs in patients with various external and/or middle ear pathological conditions.	A cross-sectional study N = 249 (male = 141; female = 108; age range: 17–60 years) Bi-atresia (N = 65); uni-atresia (N = 124); uni-stenosis + HL with normal ear canal (N = 11); uni-HL with normal ear canal (N = 13); bi-otorrhea/otitis media (N = 9); bi-HL with normal ear canal (N = 27); HL with normal ear canal (N = 13) Aided hearing thresholds in sound field Three types of the transducers were tested Speech recognition measurement using 20 monosyllables (i.e., 67-S word lists)	In the bilateral atresia group, purchase cases showed significantly better aided thresholds at 0.25 and 0.5 kHz than non-purchasers. Purchase cases in the unilateral atresia group were significantly younger than non-purchase cases. No significant differences in functional gains and SRS were found between purchase and non-purchase cases in all six groups. The continued use rate of the simple transducer was significantly lower in the otorrhea and HL with normal ear canal groups.
12. Nairn et al., 2023; USA [25]	To compare audiometric outcomes of CC-HAs with traditional BCHDs.	A prospective cohort study N = 16 (male = 9; female = 7; age range: 18–90 years) Atresia (n = 15); surgical stenosis (n = 4) Unaided/aided hearing thresholds in sound field	Mean aided pure tone averages with the BCHDs and CC-HAs were 27 and 32 dB, respectively, with a significant difference favouring BCHDs. Mean functional gains with BCHDs and CC-HAs were 54 and 49 dB, respectively, with a significant difference favouring BCHDs. Speech recognition scores were equivalent, except for the 15-dB Signal-to-Noise ratio condition, which favoured BCHDs. Pure tone audiometric outcomes with BCHDs showed a small advantage over CC-HAs, mainly via high-frequency responses. The functional gains of BCHDs were significantly higher than CC-HAs at frequencies of over 1.0 kHz.

* Note: N to indicate the number of participants, while n to represent the number of ears.

**Table 3:** Comparisons of audiological efficacy in aided patients with various ear canal pathologies.

	Atresia (n = 211 ears)	Stenosis (n = 36 ears)	Other Conditions with Normal Ear Canals (n = 23 ears)
Unaided average PTA (dB HL)	66.95 ± 13.05	65.82 ± 16.67	70.71 ± 24.05
Aided average PTA (dB HL)	32.71 ± 17.13	35.92 ± 9.29	46.29 ± 15.51
Functional gain (dB)	34.29 ± 11.59	28.71 ± 16.12 *	25.42 ± 17.62 ^#^

*: Significant increase in synchronised functional gain in the atresia group than stenosis group and patients with normal ear canals. ^#^: Significant increase in synchronised functional gain in the stenosis group than patients with normal ear canals.

## Data Availability

No new data were created or analysed in this study. Data sharing is not applicable to this article.
